# Long‐Term Prognosis of Ischemic Stroke in Young Adults—A Single‐Center Chinese Cohort Study

**DOI:** 10.1002/brb3.70479

**Published:** 2025-04-18

**Authors:** Yuhui Sha, Qiqi Wang, Mingyu Tang, Ming Yao, Yicheng Zhu, Lixin Zhou, Jun Ni

**Affiliations:** ^1^ Department of Neurology, Peking Union Medical College Hospital Peking Union Medical College and Chinese Academy of Medical Sciences Beijing China; ^2^ Department of General Medicine, Peking Union Medical College Hospital Peking Union Medical College and Chinese Academy of Medical Sciences Beijing China

**Keywords:** ischemic stroke, long‐term, prognosis, risk factors, young adults

## Abstract

**Introduction:**

Young patients with ischemic stroke often present with more complex etiologies and risk factors, making their long‐term prognosis particularly challenging. This study aims to investigate the long‐term prognosis and identify factors associated with recurrent ischemic cerebrovascular events and unfavorable functional outcome in a prospective, single‐center cohort.

**Methods:**

We consecutively enrolled young adults (aged 18–49) with ischemic stroke in the single‐center cohort at Peking Union Medical College Hospital (PUMCH) from March 2017 to March 2023. Follow‐up was conducted through face‐to‐face visits or telephone interviews. Main outcomes were recurrent ischemic cerebrovascular events and unfavorable functional outcome (an mRS score ≥ 2). Kaplan–Meier analysis was used to estimate the 5‐year cumulative recurrence risk, and multivariate logistic analysis was used to identify predictors of recurrent ischemic cerebrovascular events and unfavorable functional outcome.

**Results:**

A total of 226 patients (median (IQR) age, 35 (30–41) years; 148 male (65.5%)) were included to the final analysis. According to the TOAST classification, large‐artery atherosclerosis was identified as the most common subtype (38.1%). The 5‐year cumulative recurrence rate for ischemic cerebrovascular events was 13.5% (95% CI: 6.7%–19.9%), with no significant difference between patients with different etiologies. Low education level (OR 12.016, 95% CI: 2.805–51.469, *p* < 0.001), previous TIA (OR 9.594, 95% CI: 2.500–36.824, *p* < 0.001), previous ischemic stroke (OR 3.177, 95% CI: 1.128–8.946, *p* = 0.029), and mRS score at follow‐up (OR 3.339, 95% CI: 1.714–6.502, *p* < 0.001) were independent risk factors of ischemic cerebrovascular event recurrence after adjusting for sex and age. Baseline mRS scores was identified as an independent predictor of long‐term poor functional outcome (OR 2.264, 95%CI: 1.207–4.246, *p* = 0.011) after adjusting for sex, age, receiving antiplatelet treatment and having recurrent ischemic stroke or TIA.

**Conclusions:**

Young patients with ischemic stroke were at risk of recurrent ischemic cerebrovascular events, enhancing the need to enhance stroke prevention and treatment, particularly among young Chinese individuals with low education levels.

## Graphical abstract

The long‐term prognosis and identified risk factors associated with recurrent ischemic cerebrovascular events and unfavorable functional outcome in a single‐center cohort of young patients with ischemic stroke.

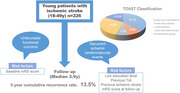

## 1 Introduction

The increasing incidence of ischemic stroke in young adults since the 1980s is a serious public health concern, particularly in regions with low to medium sociodemographic index (SDI) such as China, North Africa, the Middle East, and Southeast Asia (Zhang et al. 2023). Given the pivotal role that young adults play in familial and social structures, the consequences of ischemic stroke pose a substantial socioeconomic burden.

**Summary**

- By conducting a median 3.9‐year follow‐up of these patients, we analyzed the long‐term outcomes such as ischemic cerebrovascular event recurrence and neurological function, as well as their predictive factors.
- The study indicated that the 5‐year cumulative recurrence rate of cerebrovascular events among young stroke patients is 13.5%.

Despite having more complicated etiologies and risk factors, young patients with ischemic stroke generally experience more favorable short‐term outcomes compared to stroke in the elderly (Brouwer et al. 2022; Fonarow et al. 2010). However, the long‐term prognosis of young stroke survivors is often challenging due to many potential complications. Recent epidemiological studies have shown that young patients with ischemic stroke have a fivefold higher long‐term mortality rate compared to the control population (Ekker et al. 2019). Multiple health and socioeconomic consequences include long‐term risk of recurrent stroke (Verburgt et al. 2024), physical disability (Synhaeve et al. 2014), cognitive impairment (Schaapsmeerders et al. 2013), and difficulty returning to work (Aarnio et al. 2018), which can also seriously affect quality of life (Naess et al. 2012). The relative scarcity of such data in China is a concerning gap. Therefore, attention and tailored interventions are necessary to mitigate this escalating health issue. The goal is not only to reduce the risk of stroke recurrence, but also to ensure that those affected can successfully reintegrate into society after recovery.

The aim of this study is to investigate the long‐term prognosis and identity factors associated with recurrent ischemic stroke or TIA and unfavorable functional outcome in a prospective single center cohort study on ischemic stroke in young adults, so as to provide a basis for formulating targeted prevention and treatment strategies and further improve the prognosis of young stroke in China.

## 2 Materials and Methods

### 2.1 Study Population

**FIGURE 1:**
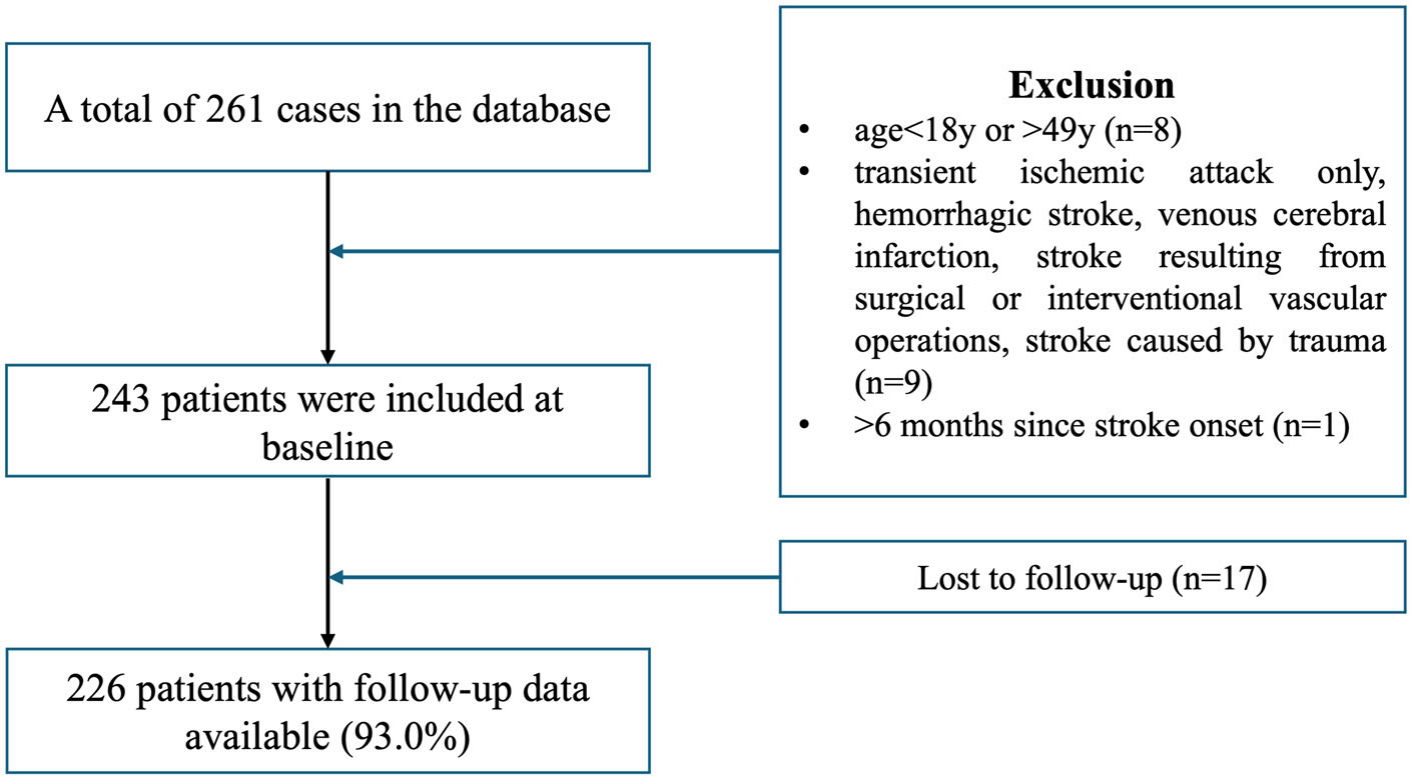
Flowchart of the patient inclusion in the study.

We consecutively enrolled young adults with ischemic stroke in the single‐center prospective observational Young Stroke Etiology and Risk Factor Cohort at Peking Union Medical College Hospital (PUMCH) from March 2017 to March 2023 in this cross‐sectional follow‐up study. The inclusion criteria were age of onset 18–49 years, having an acute ischemic stroke within the last 6 months, and providing written informed consent. Exclusion criteria were transient ischemic attack (TIA) only, intracerebral hemorrhage, cerebral venous sinus thrombosis, stroke resulting from surgical or interventional vascular operations (such as carotid endarterectomy, stenting, or cardiac surgery), and stroke caused by trauma (Figure 1).

This study adhered to the Strengthening the Reporting of Observational Studies in Epidemiology (STROBE) reporting guideline and was approved by the Ethics Committee of PUMCH (reference numbers: JS‐1281). All patients provided written informed consent following a comprehensive explanation of the research protocol.

### 2.2 Study Design

Baseline characteristics included demographic characteristics, etiologies, baseline disability caused by index stroke, medical history and risk factors, and medication use.

Demographic characteristics included age, sex, ethnicity, education level, and occupation. Low education level was defined as having ≤ 6 years of education (primary school or illiterate) (Synhaeve et al. 2014). Medical history and risk factors included a history of ischemic stroke, TIA, hypertension, diabetes mellitus, hyperlipidemia, coronary heart disease, atrial fibrillation/flutter, smoking, alcohol consumption, patent foramen ovale, migraine, pregnancy/postpartum, and long‐term oral contraceptives. Family history of early‐onset stroke was defined as a history of cerebrovascular disease among first‐ and second‐degree relatives with an onset age of 18–49 years. Traditional vascular risk factors referred to a history of hypertension, diabetes mellitus, hyperlipidemia, smoking, coronary heart disease, and family history of early‐onset stroke. Baseline disability caused by index stroke was assessed using the modified Ranking Scale (mRS) score at the time of inclusion. The etiologies of ischemic stroke were classified using Trial of Org 10172 in Acute Stroke Treatment (TOAST) classification, including large artery atherosclerosis, small vessel occlusion, cardioembolism, other determined etiology, and undetermined etiology. The other determined etiologies included Moyamoya disease, arterial dissection, systemic lupus erythematosus, antiphospholipid syndrome, vasculitis, eosinophilia, eosinophilic granulomatous vasculitis, fibromuscular dysplasia, deficiency of adenosine deaminase 2, paroxysmal nocturnal hemoglobinuria, hyperhomocysteinemia, hemolytic uremic syndrome, etc. The medication use included antithrombotic drugs (antiplatelet therapy, anticoagulation therapy), statins, and antihypertensive therapy.

### 2.3 Follow‐Up

Recurrent events and other prognosis were collected through standardized, structured questionnaires by face‐to‐face follow‐up between Aug 2023 and March 2024. Telephone follow‐ups were conducted if patients were unable to visit the hospital. Follow‐up assessments included recurrent ischemic stroke or TIA, death, mRS score, medication use, and medication adherence. Recurrent ischemic stroke or TIA was defined based on medical records, including neurological manifestations and MRI findings, as diagnosed by experienced physicians.

The medication uses at follow‐up included antithrombotic drugs (antiplatelet therapy, anticoagulation therapy), statins, and antihypertensive therapy. The medication adherence was assessed by the Morisky Medication Adherence Scale 8‐item (MMAS‐8) (Yan et al. 2014). Events, the assessment scales and medication use are classified by trained assessors and judged by experts in the corresponding field.

### 2.4 Main Outcome

The main outcomes were recurrent ischemic stroke or TIA and unfavorable functional outcome, defined as an mRS score≥2 at follow‐up, respectively.

### 2.5 Statistical Analysis

Continuous variables were described using *Median (IQR)* considering their skewed distribution. Categorical variables were expressed as frequency *N (%)*. The Mann–Whitney *U‐*test was used to compare continuous variables between the two groups and Pearson's *χ* ^2^ *test* was used for categorical variables. The 5‐year cumulative recurrence rate was calculated using the Kaplan–Meier survival curve method.

Multivariate logistic regression analysis was performed to evaluate predictors for recurrent ischemic stroke or TIA, neurological outcomes and return‐to‐work status in young patients with ischemic stroke. Patients were initially divided into two groups based on the three outcome measures mentioned above respectively. Univariate analysis was conducted by the Mann–Whitney *U‐*test or Pearson's *χ* ^2^ *test* to identify factors with significant difference between the two groups. Variables with statistical significance from the univariate analysis were then included as covariates in the multivariable logistic regression models (using enter selection procedures).

Statistical significance was defined as a two‐tailed *p < 0.05*. Statistical analyses were conducted using SPSS 26.0 and *R studio*.

## 3 Results

In total, 243 young patients with ischemic stroke were included in the study. Seventeen patients (7.0%) were lost to follow‐up. The median follow‐up period was 3.9 (IQR: 2.6, 4.8) years. Finally, a total of 226 patients (median (IQR) age, 35 (30–41) years; 78 female (34.5%); 148 male (65.5%)) were included to the final analysis.

Table 1 shows the characteristics of the patients. The median baseline mRS score was 1 (IQR: 0, 1). At least one traditional risk factor was found in 179 (79.2%) patients. Fifty‐nine (26.1%) patients had previous ischemic stroke and 21 patients (9.3%) had previous TIA. According to the TOAST classification, large‐artery atherosclerosis was identified as the most common subtype (38.1%), followed by other determined etiology (28.8%), undetermined etiology (16.8%), small‐vessel occlusion (8.8%), and cardioembolism (7.5%). In terms of treatment for ischemic stroke, 200 patients (88.5%) received antiplatelet treatment, 17 patients (7.5%) received anticoagulation treatment, and 204 patients (90.3%) used statins.

**TABLE 1:** Univariate and multivariate analysis of recurrent ischemic stroke or TIA in young patients with ischemic stroke.

		Univariate analysis			Multivariate analysis	
	All *n* = 226	Without recurrent ischemic stroke or TIA	With recurrent ischemic stroke or TIA	*p*	Odds ratio (95%CI)	*p*
		*N* (%) / Median (IQR)	*N* (%) / Median (IQR)			
		*n* = 201	*n* = 25			
**Demographic**						
Sex (female)	78 (34.5%)	67 (33.3%)	11 (44.0%)	0.290	2.366 (0.836, 6.696)	0.105
Age (years)	35 (30, 41)	35 (30, 41)	35 (31, 38)	0.428	0.929 (0.863, 0.999)	0.050
Low education level	21 (9.3%)	15 (7.5%)	6 (24.0%)	**0.020**	12.016 (2.805, 51.469)	**< 0.001**
**TOAST classification**				0.305		
Large artery atherosclerosis	86 (38.1%)	78 (38.8%)	8 (32.0%)			
Small vessel occlusion	20 (8.8%)	18 (9.0%)	2 (8.0%)			
Cardioembolism	17 (7.5%)	16 (8.0%)	1 (4.0%)			
Other determined etiology	65 (28.8%)	59 (29.4%)	6 (24.0%)			
Undetermined etiology	38 (16.8%)	30 (14.9%)	8 (32.0%)			
**Baseline mRS score (at the time of inclusion)**	1 (0,1)	1 (0, 1)	1 (0, 1)	0.197		
**Traditional vascular risk factors**						
Hypertension	68 (30.1%)	64 (31.8%)	4 (16.0%)	0.103		
Diabetes mellitus	23 (10.2%)	20 (10.0%)	3 (12.0%)	1.000		
Smoking	95 (42.0%)	88 (43.8%)	7 (28.0%)	0.132		
Hyperlipidemia	61 (27.0%)	55 (27.4%)	6 (24.0%)	0.721		
Coronary heart disease	3 (1.3%)	2 (1.0%)	1 (4.0%)	0.298		
Family history of early‐onset stroke	33 (14.6%)	27 (13.4%)	6 (24.0%)	0.267		
≥1 traditional vascular risk factors	179 (79.2%)	160 (79.6%)	19 (76.0%)	0.676		
**Other vascular risk factors**						
History of ischemic stroke	59 (26.1%)	47 (23.4%)	12 (48.0%)	**0.008**	3.177 (1.128, 8.946)	**0.029**
History of TIA	21 (9.3%)	15 (7.5%)	6 (24.0%)	**0.020**	9.594 (2.500, 36.824)	**< 0.001**
Alcohol	75 (33.2%)	68 (33.8%)	7 (28.0%)	0.559		
Atrial fibrillation/flutter	3 (1.3%)	2 (1.0%)	1 (4.0%)	0.298		
Migraine	10 (4.4%)	9 (4.5%)	1 (4.0%)	1.000		
Patent foramen ovale	6 (2.7%)	4 (2.0%)	2 (8.0%)	0.134		
Long‐term oral contraceptives	2 (0.9%)	2 (1.0%)	0	0.791		
**Treatment at follow‐up**						
Antiplatelet therapy	200 (88.5%)	180 (90.0%)	20 (80.0%)	0.280		
Anticoagulation therapy	17 (7.5%)	14 (7.0%)	3 (12.0%)	0.618		
Statins	204 (90.3%)	181 (90.0%)	23 (92.0%)	1.000		
Antihypertensive therapy	54 (23.9%)	50 (24.9%)	4 (16.0%)	0.326		
**Medication adherence**	7 (6, 7.38)	7 (6, 7.75)	7 (7, 7.75)	0.241		
**mRS score at follow‐up**	1 (0, 1)	0 (0, 1)	1 (0, 1)	**0.004**	3.339 (1.714, 6.502)	**< 0.001**

Values in bold font indicate statistically significant differences at p < 0.05.

### 3.1 Characteristics and Predictors of Recurrent Ischemic Stroke or TIA in Young Patients With Ischemic Stroke

During the follow‐up of 3.9 years, 25 (11%) patients experienced recurrent ischemic stroke or TIA, including 15 with ischemic stroke and 10 with TIA. The median age of patients with recurrent ischemic stroke or TIA was 35 years (IQR: 31, 38), with 44% being female. The most common etiologies of the recurrent ischemic stroke or TIA were LAA and undetermined etiology, each accounting for 32.0%.

**FIGURE 2:**
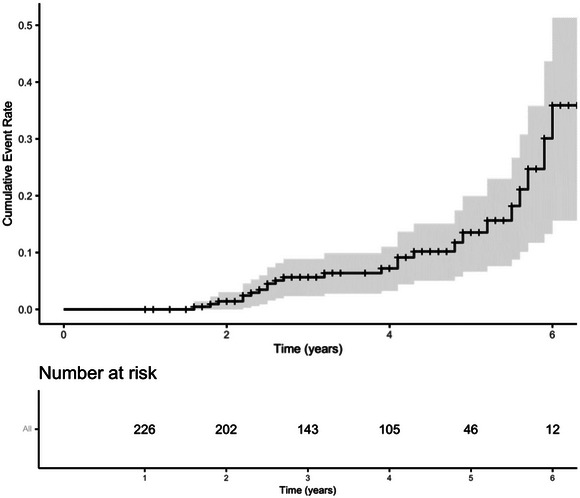
Cumulative risks and numbers of patients at risk for recurrent ischemic cerebrovascular event.

**FIGURE 3:**
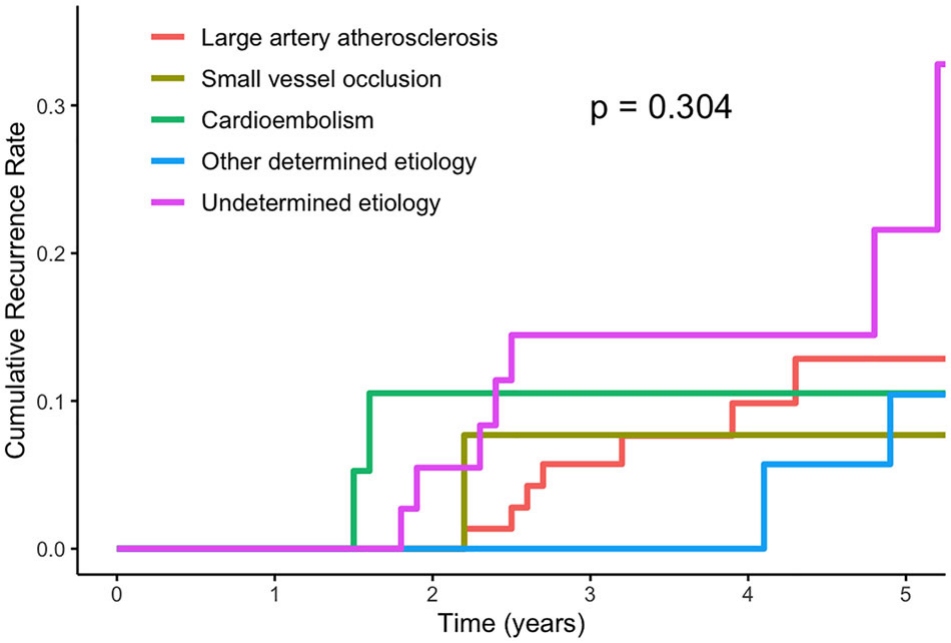
Cumulative risk of recurrent ischemic cerebrovascular event stratified by stroke subtype (modified Trial of Org 10172 in Acute Stroke Treatment (TOAST) criteria) (*p* = 0.304).

The 5‐year cumulative incidence was 13.5% (95% CI: 6.7%–19.9%) for recurrent ischemic stroke or TIA as calculated by Kaplan–Meier survival curve method (Figure 2). For patients with different cause of stroke according to TOAST classification, the 5‐year cumulative recurrence rates were 21.6% (95% CI: 2.4%–37%) for undetermined etiology, followed by 12.9% (95% CI: 2.9%–21.8%) for large‐artery atherosclerosis, 10.5% (95% CI: 0–23.3%) for small‐vessel occlusion, 10.4% (95% CI: 0–21.3%) for other determined etiology, and 7.7% (95% CI: 0–21.1%) of cardioembolism, with no significant difference between patients with different etiologies (*p* = 0.304) (Figure 3).

Compared to patients without recurrent ischemic stroke or TIA, patients with recurrent ischemic stroke or TIA had a higher proportion of individuals with low education levels (24.0% vs. 7.5%, *p* = 0.020), previous TIA (24.0% vs. 7.5%, *p* = 0.020), and previous ischemic stroke (48.0% vs. 23.4%, *p* = 0.008). The follow‐up mRS score was significantly higher in patients with recurrent ischemic stroke or TIA (1 vs. 0, *p* = 0.004). There was no significant difference of sex, age, TOAST classification, initial mRS score, traditional risk factors, other vascular risk factors, treatment conditions, and medication adherence between the two groups.

Logistic multivariate regression analysis showed that low education level (OR 12.016, 95% CI: 2.805–51.469, *p* < 0.001), previous TIA (OR 9.594, 95% CI: 2.500–36.824, *p* < 0.001), previous ischemic stroke (OR 3.177, 95% CI: 1.128–8.946, *p* = 0.029), and mRS score at follow‐up (OR 3.339, 95% CI: 1.714–6.502, *p* < 0.001) were independent risk factors of ischemic cerebrovascular event recurrence, after adjusting for sex and age (Table 1).

### 3.2 Characteristics and Predictors of Unfavorable Functional Outcome (mRS ≥2) in Young Patients With Ischemic Stroke

Long‐term functional independence (mRS < 2) was observed in 212 patients (93.8%). Among the 14 patients with unfavorable functional outcome (mRS > = 2), the median age was 40 years (IQR: 30.5, 42.75) and 42.9% were female, with LAA and other determined etiology accounting for 35.7% and 28.6% respectively. Compared to patients with functional independence, those with unfavorable functional outcome had higher baseline mRS score (1 (IQR: 1, 4) vs. 1 (IQR: 0, 1), *p* = 0.036), lower proportion of receiving antiplatelet therapy (64.3% vs. 90.1%, *p* < 0.001), and higher prevalence of recurrent ischemic stroke or TIA (35.7% vs. 9.4%, *p* = 0.009). There was no significant difference in sex, age, traditional vascular risk factors, other vascular risk factors, etiologies, and medication adherence between patients with and without unfavorable functional outcomes.

Baseline mRS scores was identified as an independent predictor of long‐term poor functional outcome in the multivariate analysis (OR 2.264, 95%CI: 1.207–4.246, *p* = 0.011), after adjustment for sex, age, receiving antiplatelet treatment and having recurrent ischemic stroke or TIA (Table 2).

**TABLE 2:** Univariate and multivariate analysis of unfavorable functional outcome in young patients with ischemic stroke.

	Univariate analysis			Multivariate analysis	
	Follow‐up mRS < 2	Follow‐up mRS ≥ 2	*p*	Odds ratio (95%CI)	*p*
	*N* (%) / Median (IQR)	*N* (%) / Median (IQR)			
	*n* = 212	*n* = 14			
**Demographic**					
Sex (female)	72 (34.0%)	6 (42.9%)	0.120	1.091 (0.249, 4.775)	0.908
Age (years)	35 (30,41)	40 (30.5, 42.75)	0.138	1.045 (0.943, 1.158)	0.402
Low education level	19 (9.0%)	2 (14.3%)	0.850		
**TOAST classification**			0.992		
Large artery atherosclerosis	81 (38.2%)	5 (35.7%)			
Small vessel occlusion	19 (9.0%)	1 (7.1%)			
Cardioembolism	16 (7.5%)	1 (7.1%)			
Other determined etiology	61 (28.8%)	4 (28.6%)			
Undetermined etiology	35 (16.5%)	3 (21.4%)			
**Baseline mRS score (at the time of inclusion)**	1 (0, 1)	1 (1, 4)	**0.036**	2.264 (1.207, 4.246)	**0.011**
**Traditional vascular risk factors**					
Hypertension	65 (30.7%)	3 (21.4%)	0.668		
Diabetes mellitus	22 (10.4%)	1 (7.1%)	1.000		
Smoking	92 (43.4%)	3 (21.4%)	0.107		
Hyperlipidemia	58 (27.4%)	3 (21.4%)	0.862		
Coronary heart disease	3 (1.4%)	0	1.000		
Family history of early‐onset stroke	32 (15.1%)	1 (7.1%)	0.671		
≥1 traditional vascular risk factors	168 (79.2%)	11 (78.6%)	1.000		
**Other vascular risk factors**					
History of ischemic stroke	54 (25.5%)	5 (35.7%)	0.595		
History of TIA	21 (9.9%)	0	0.447		
Alcohol	71 (33.5%)	4 (28.6%)	0.932		
Atrial fibrillation/flutter	2 (0.9%)	1 (7.1%)	0.175		
Migraine	10 (4.7%)	0	1.000		
Patent foramen ovale	5 (2.4%)	1 (7.1%)	0.322		
Long‐term oral contraceptives	2 (0.9%)	0	0.880		
**Treatment at follow‐up**					
Antiplatelet therapy	191 (90.1%)	9 (64.3%)	**0.012**	0.239 (0.046, 1.240)	0.088
Anticoagulation therapy	14 (6.6%)	3 (21.4%)	0.130		
Statins	193 (91.0%)	11 (78.6%)	0.290		
Antihypertensive therapy	50 (23.6%)	4 (28.6%)	0.920		
**Medication adherence**	7 (6, 7.5)	7.25 (7, 8)	0.155		
**Recurrent ischemic stroke or TIA**	20 (9.4%)	5 (35.7%)	**0**.**009**	5.540 (0.900, 34.116)	0.065

Values in bold font indicate statistically significant differences at p < 0.05.

## 4 Discussion

This study is a cross‐sectional follow‐up of the long‐term prognosis in young ischemic stroke patients of China. By conducting a median 3.9‐year follow‐up of these patients, we analyzed the long‐term outcomes such as ischemic cerebrovascular event recurrence and neurological function, as well as their predictive factors. The study indicated that the 5‐year cumulative recurrence rate of cerebrovascular events among Chinese young stroke patients is 13.5%. Low educational level and a history of previous stroke and TIA significantly increased the risk of recurrence of ischemic stroke. The long‐term functional prognosis of young patients with ischemic stroke is generally favorable, with poor prognosis significantly associated with the severity of disability caused by the index stroke.

This study found that the 5‐year cumulative stroke recurrence rate among young ischemic stroke patients in China is 13.5% (95% CI: 6.7%–19.9%), which is generally consistent with those reported in other young stroke cohorts. A multicenter prospective cohort study in Netherland, which included 1,216 patients with a median follow‐up of 4.3 years, found the 5‐year risk was 12.2% (Verburgt et al. 2024). Similarly, a large cohort study following 807 young stroke patients for 5 years found a cumulative 5‐year recurrence rate of 9.4% for ischemic stroke (Putaala et al. 2010). Regarding the etiologies classified by the TOAST criteria, most studies have identified large‐artery atherosclerosis as having the highest cumulative risk of recurrence (22.7%–24.7%) (Pezzini et al. 2014; Putaala et al. 2010; Verburgt et al. 2024; Verhoeven et al. 2022). However, in this study, we did not find a significant difference in the 5‐year cumulative recurrence risk among the different TOAST subtypes, probably due to the small sample size. We found that patients with cryptogenic stroke had the highest cumulative recurrence risk, exceeding those with large‐artery atherosclerosis. This might be attributed to most patients with large‐artery atherosclerosis in our study received standard secondary prevention, including antiplatelet therapy, intensive lipid‐lowering treatment, and active management of risk factors, which together reduced the recurrent risk. The diagnosis of cryptogenic stroke in this cohort was made through comprehensive and thorough evaluations, enhancing diagnostic accuracy. The high recurrence risk in patients with undetermined etiology can be attributed to several factors. First, etiology‐specific treatment strategies for patients with cryptogenic stroke remained unclear, and most patients were only treated with empirical antiplatelet therapy. Besides, it was difficult to identify and manage all potential risk factors in these patients. Additionally, the optimal duration of antithrombotic therapy was uncertain, leading some patients to discontinue secondary prevention at early time. We have to acknowledge that the follow‐up period may not have been long enough to fully capture the long‐term prognosis. Data on recurrence rates among young stroke patients in Asia have been limited and our study contributes valuable insight into the recurrence of ischemic stroke in this population.

Previous studies have identified risk factors for stroke recurrence in young adults, including TOAST etiology of large artery atherosclerosis, cardioembolism (Rutten‐Jacobs et al. 2013; Verhoeven et al. 2022) and lacunar stroke (Rutten‐Jacobs et al. 2013), diabetes mellitus (Verburgt et al. 2024; Verhoeven et al. 2022), atrial fibrillation (Broman et al. 2023; Wang et al. 2022), age ≥ 45 years (Wang et al. 2022), and alcohol abuse (Verburgt et al. 2024). A study evaluating young adults with embolic stroke of undetermined source found that stroke or transient ischemic attack, presence of diabetes, and a history of coronary artery disease were predictors of recurrent ischemic stroke (Perera et al. 2022). In our study, we found that a lower educational level and a history of stroke and TIA are significant predictors for stroke recurrence in young adults, whereas stroke etiology, traditional vascular risk factors, or treatment conditions showed no significant association with recurrence. Previous research has consistently demonstrated a strong correlation between educational level and stroke prognosis, with patients of lower socioeconomic status being more susceptible to stroke recurrence and poorer functional outcomes (Che et al. 2020). Our study observed similar findings in young patients with ischemic stroke in China, highlighting the urgency of continuous monitoring and follow‐up of this population due to their socioeconomic disadvantage. In addition, we emphasize the necessity of improving medication adherence education in order to effectively enhance patients' quality of life and prognosis. Furthermore, our study identified a significant association between stroke recurrence and long‐term poor functional outcomes. A longitudinal study on young ischemic stroke found that recurrent vascular events were associated with poor functional outcome in patients with diabetes (Shareef et al. 2024). The correlation between stroke recurrence and functional outcome has been well‐established. One study found that stroke recurrence significantly increased the risk of disability at 5 years (Hobeanu et al. 2022). These findings emphasized the importance of rigorous patient management and secondary prevention in improving the prognosis of young stroke patients. In our multivariate regression analysis, we did not observe significant association between stroke etiology or modifiable cerebrovascular risk factors and recurrent ischemic events. This may be partly attributed to the relatively small sample size of our study, as well as the possibility that patients in our cohort received standardized etiological diagnosis and secondary prevention.

Research on the long‐term functional outcome of young ischemic stroke patients has been limited. One study including 287 young adults with ischemic stroke with a median follow‐up of 3 years reported that 86.8% of them achieved a follow‐up mRS ≤ 2 (Leys et al. 2002). Another prospective study with a median follow‐up of 9.1 years found that 32% of all young ischemic stroke patients had a follow‐up mRS > 2 (Synhaeve et al. 2014). Another study on ischemic stroke in young adults found 76.7% of all patients showed no or minimal clinical deficit (mRS < 2) (Spengos and Vemmos 2010). In our study, 93.8% of young ischemic stroke patients exhibited favorable neurological outcome (mRS < 2). The high proportion of patients with favorable outcome in our cohort could be explained by the following reasons. Firstly, most patients in our cohort received standardized etiological diagnosis process, targeted treatment and secondary prevention. Secondly, as our cohort primarily focused on investigating the etiology and risk factors of young stroke, we may have included a higher proportion of non‐disabling patients at baseline, introducing the selection bias. Additionally, the overall age of our study population was younger than that of similar cohorts (Synhaeve et al. 2014), possibly contributing to relatively better outcomes. Moreover, the follow‐up period may be not long enough to capture more adverse outcomes. In this study, baseline disability caused by the index stroke was an independent risk factor for poor outcomes at follow‐up. This finding aligns with other studies that identified the NIHSS score at admission as an independent predictor of unfavorable functional outcomes (Synhaeve et al. 2014; Wang et al. 2022). In the univariate analysis, patients with unfavorable outcome had a lower proportion of receiving antiplatelet therapy and a higher rate of recurrent ischemic events. These findings emphasized the importance of standardized antithrombotic therapy and secondary prevention in improving the prognosis of young stroke patients. Further studies with larger sample sizes and extended follow‐up periods are necessary to obtain comprehensive data on the long‐term outcomes and influencing factors for young ischemic stroke patients in China.

We propose the following strategies to improve the identification of unusual stroke etiologies in young patients. Clinically, optimizing neuroimaging protocols, particularly through high‐resolution vessel wall MRI and ultra‐high‐field MRI (5.0T/7.0T), is essential to characterize specific vascular pathologies such as branch atheromatous disease and moyamoya syndrome. Concurrently, implementing a systematic diagnostic framework should integrate evaluations for autoimmune, infectious, inflammatory, hematologic, metabolic or malignant disorders. Medication adherence, perioperative risks, drug‐induced mechanisms must also be rigorously assessed. For cryptogenic cases, targeted genetic testing should be considered especially in patients with familial predisposition or atypical phenotypes. Advancing multiomics studies to explore genomic signatures and novel biomarkers in young stroke cohorts could elucidate pathophysiological pathways and refine risk stratification. Collaborative efforts to establish large‐scale phenotype‐genotype databases will further accelerate the identification of rare stroke subtypes and inform precision prevention strategies.

This study has some limitations that need to be addressed. First, it is a single‐center cohort from a large tertiary hospital which focused on the investigation of etiology and risk factors, and given the presence of loss to follow‐up, there is a certain degree of selection bias. In addition, the follow‐up period is relatively short with varying follow‐up duration, which may limit the generalizability of the findings. Also, we used mRS instead of NIHSS score at baseline because some patients with ischemic stroke were not in the acute phase at the time of enrollment. Future research should include a larger sample size and extend the follow‐up period to enable more comprehensive statistical analysis.

## 5 Conclusions

In conclusion, our study found that the 5‐year cumulative incidence for recurrent ischemic stroke or TIA among young adults with ischemic stroke is 13.5% in China. It was identified that factors including low education level, a history of ischemic stroke or TIA significantly increased the risk of ischemic stroke recurrence. While young patients with ischemic stroke generally demonstrated favorable long‐term functional prognosis, a poor outcome is significantly associated with the baseline disability caused by index stroke. These findings highlight the need to enhance stroke prevention and acute treatment, particularly among young individuals with low education levels in China. Future research should include larger sample sizes and extended follow‐up periods to provide more comprehensive prognostic data for young stroke patients, and develop more precise prevention and treatment strategies to improve the outcomes of them.

## Author Contributions

**Yuhui Sha**: writing–original draft, formal analysis, data curation, investigation. **Qiqi Wang**: writing–original draft, data curation, formal analysis, investigation. **Mingyu Tang**: data curation, investigation. **Ming Yao**: data curation. **Yicheng Zhu**: data curation. **Lixin Zhou**: conceptualization, writing–review and editing, supervision. **Jun Ni**: writing–review and editing, conceptualization, supervision.

## Conflicts of Interest

The authors declare that they have no competing interests.

### Ethics Statement

This study was conducted in accordance with the Declaration of Helsinki and approved by the ethical review board of PUMCH (reference numbers: JS‐1281). All patients have signed informed consent forms.

### Peer Review

The peer review history for this article is available at https://publons.com/publon/10.1002/brb3.70479

## Data Availability

Anonymized data associated with the research presented will be made available from the corresponding author upon reasonable request.
